# Age exacerbates the negative effect of depression on executive functioning in racial and ethnic minorities

**DOI:** 10.1007/s11682-024-00898-3

**Published:** 2024-06-08

**Authors:** Zhimei Niu, Andreana P. Haley, Alexandra L. Clark, Audrey Duarte

**Affiliations:** Department of Psychology, University of Texas at Austin, 108 Dean Keeton Street, Austin, TX 78712 USA

**Keywords:** Aging, Depression, Ethnoracial diversity, Cognitive impairment, Prefrontal cortex volumes

## Abstract

Age and depression may interact to produce a “double jeopardy” for cognitive impairment, and executive functioning, in cognitively unimpaired aging. Few studies have considered middle age or the ethnoracial diversity of subjects, despite evidence of more severe cognitive outcomes in historically minoritized people. In this pilot study, we investigated the impact of age on depression-related cognitive impairment and the underlying brain volumes in middle-aged non-Hispanic White adults (116), and Hispanic and Black adults (60), with a total number of 176 adults. The result shows a significant interaction between age and depression for executive functioning, specifically for middle-aged Hispanic and Black adults, but not non-Hispanic White adults. Prefrontal cortex volumes, which were reduced in the Black and Hispanic compared to the non-Hispanic White adults, partially mediated the relationship between depression level and executive functioning, across age and ethnoracial group. Collectively, these results suggest that the negative impact of depression on executive functioning and Prefrontal cortex volumes integrity may be exacerbated by age and that historically minoritized people may be particularly sensitive to this double jeopardy.

**Supplementary Information:**

The online version contains supplementary material available at 10.1007/s11682-024-00898-3.

## Introduction

Major depressive disorder (MDD) is highly prevalent across an adult’s lifetime with a risk rate of 15–18% (Malhi & Mann, 2018). Evidence shows a increase rate of MDD from adolescence with relative stability through middle age (Goodwin et al., 2022). Adults over the age of 55 are less likely to experience MDD than younger individuals but are more likely to experience sub-threshold depressive symptoms (Biella et al., 2019). Both MDD and sub-threshold depressive symptoms not only negatively impact quality of life, but also may contribute to impairments in cognitive functioning, perhaps most notably in executive functioning (Lam et al., 2014; Rock et al., 2014; Rutherford et al., 2023; Wu et al., 2019). Importantly, greater levels of depressive symptomology in older adults are associated with an increased risk of developing dementia (Morimoto et al., 2014). Thus, it is important to identify the mechanisms of depression-associated cognitive impairment earlier in the adult lifespan for the future development of interventions that can delay or prevent cognitive decline. Neuroimaging studies are particularly important for elucidating neurobiological mechanisms that contribute to both mood and cognitive dysfunction that may serve as targets for future pharmacological and non-pharmacological interventions.

There are several reasons why age may exacerbate depression-related impairments in executive functioning. Both aging(e.g., Campbell et al., 2012; Dulas & Duarte, 2016; Rey-Mermet et al., 2018) and depression are independently associated with executive dysfunction as measured by a number of neuropsychological tests (i.e. Trail Making Test, Stroop Color-Word Interference Test, Wisconsin Card Sorting Test (WCST), across the adult lifespan. Furthermore, both age and depression are independently associated with reduced prefrontal cortex (PFC) volumes in regions supporting executive functioning.(Dulas & Duarte, 2016; Grahek et al., 2018, 2019; Yang et al., 2017; Zaninotto et al., 2016) For example, adults with MDD, compared to non-depressed adults, show reduced volume in multiple brain areas, most notably, right inferior frontal gyrus, dorsolateral prefrontal cortex (dlPFC), lateral orbital frontal cortex (lOFC), and anterior cingulate cortex (ACC) across age (Grieve et al., 2013; Niida et al., 2019; Pizzagalli & Roberts, 2022; Van Tol et al., 2010; Wigmore et al., 2017; Zhang et al., 2018). Even sub-threshold depressive symptoms in middle-aged and older adults are associated with smaller OFC and ACC volumes(Dotson et al., 2009). Given the importance of these regions in executive functioning as shown in numerous fMRI and patient studies(Pizzagalli & Roberts, 2022), such reductions could contribute to the cognitive deficits observed in both depression and aging. Finally, as shown in a recent meta-analysis, depression-related executive dysfunction in both MDD and subthreshold depression is magnified by age(Dotson et al., 2020). Collectively, these results suggest that higher levels of depression may move forward the point at which cognitive impairments characteristic of aging appear, in other words, cognitive impairment appears earlier in depressed individuals.

Few studies have assessed depression-related executive dysfunction in historically minoritized people despite evidence of racial and ethnic differences in depressive symptom prevalence. Black and Hispanic adults have a lower lifetime prevalence of diagnosed MDD but higher prevalence of sub-threshold depressive symptoms than Non-Hispanic Whites adults(Abrams & Mehta, 2019; Barnes & Bates, 2017). In both Hispanic and Black adults, symptoms may increase and be more severe in the mid-to-older adult age range when compared to non-Hispanic White adults in the same age range (Abrams & Mehta, 2019; Vyas et al., 2020), and episodes of depression tend to be more severe, persistent, and disabling (e.g., Breslau et al., 2006; Williams et al., 2007). Furthermore, depressive symptoms tend to be prolonged and associated with more severe consequences in Black adults like higher levels of distress and higher rates of treatment drop-out(Bailey et al., 2019; Barnes & Bates, 2017). Few studies have examined depression-related cognitive impairments in these racially/ethnically minoritized groups but recent evidence shows that older (> 65) Hispanic adults with depression are more susceptible to cognitive impairment than older non-Hispanic White adults (Babulal et al., 2022). Given prior evidence that age may exacerbate depression-related cognitive impairments in samples consisting largely non-Hispanic White adults (Dotson et al., 2009; James & Duarte, 2023), it is critical to understand whether such “double jeopardy” effects are also observed in historically racially/ethnically minoritized adults and the underlying neural mechanisms of these impairments.

This pilot study aims to examine (1) the potential mediating role of PFC volumes in the relationship between depressive symptoms and executive functioning and; (2) the moderating influence of age on these associations in historically minoritized, specifically Black and Hispanic adults, and non-Hispanic White middle-aged adults. We predicted that individuals with greater levels of depressive symptoms would show lower executive functioning and smaller lateral, orbital, and/or ACC frontal volumes, with PFC volumes mediating associations between depression and executive functioning. Finally, we explored the possibility that depression-cognition associations differ between ethnoracial minorities and non-Hispanic White adults, with potentially stronger associations in racial/ethnic minority groups given some recent evidence (Babulal et al., 2022; Hokett et al., 2022). We tested the possibility that age would exacerbate these associations more for these historically minoritized groups than non-Hispanic White adults (i.e., “double jeopardy”).

## Methods

### Participants

We enrolled 257 individuals between the ages of 40–61. This sample includes some of the adults whose data were published in a prior study (Foret et al., 2021). All participants self-reported that they were fluent English speakers, with normal or corrected to normal vision, and right-handed. None of the participants reported a history of neurological disease or disorders, major psychiatric illness (i.e., Schizophrenia, bipolar disorder, MDD), history of substance abuse, or MRI contraindications. Of the 257 participants enrolled, we excluded individuals missing education or incomplete cognitive and BDI scores. In total 81 participants were excluded, which includes 33 with missing MRI data. The remaining 48 individuals were excluded due to missing educational data, age, race or incomplete cognitive and BDI score: in regard to missing data at the outset, 15 missing age, 14 missing cognitive scores, 2 missing BDI score, 3 missing both cognitive and BDI score, 1 missing cognitive score and data for years of education, 3 missing data for years of education, 10 missing more than one of these variables. Of the remaining 176 participants, participants are self-identified as Non-Hispanic White adults (116), Hispanic or Black American adults (60). We collapsed across Hispanic and Black participants for the subsequent analyses to increase power. Demographic characteristics of each group are shown in Table 1.

**Table 1:** Demographic characteristics of subjects. Group differences were evaluated with two-sample *t*-tests

Group	Non-Hispanic white adults	Black & Hispanic adults
Number of subjects	116	60
Age	49.59 ± 6.39	49.1 ± 6.56
Sex (male/female)	89(M)/104(F)	37(M)/24(F)
BDI (mean)	7.31 ± 6.55	7.28 ± 7.23
Years of education	16.68 ± 2.4*	15.68 ± 2.56
Executive Function(z-scored)	-0.16 ± 0.91*	0.35 ± 1.39
WASI vocab	48.47 ± 7.77*	42.38 ± 7.96
WASI matrix	22.36 ± 3.88*	19.43 ± 4.34
COWAT total	44.96 ± 11.07*	37.7 ± 11.56
Digit Span Backward	9.26 ± 2.52*	8.25 ± 2.03
BMI	28.57 ± 7	29.55 ± 6.08
LPFC volume (ml)	45.66 ± 4.03*	43.48 ± 4.25
OFC volume (ml)	22.66 ± 2.11*	21.07 ± 1.93
ACC volume (ml)	9.56 ± 1.08*	8.77 ± 1.04
ICV(L)	1.45 ± 0.13*	1.35 ± 0.15

*Note* Means shown with standard deviation, *indicates *p* < 0.05

### Neuropsychological assessments

All subjects provided written informed consent approved by the U.T Austin Institutional Review Board. Subjects provided a self-reported medical history, the Beck Depression Index II (BDI-II) (Beck et al., 1996), and were given a battery of neuropsychological tests to assess executive functioning and other cognitive domains: Trail Making Test (Reitan & Reitan, 1992), California Verbal Learning Test II (CVLT-II) (Delis et al., 2000), Digit span, vocab and matrix subtests of the Wechsler Adult Intelligence Scale, 3rd edition (WAIS-III) (Wechsler, 1997), controlled oral word association test (COWAT) total score from multilingual aphasia examination (MAE) (Benton et al., 1994). The BDI-II has been validated for assessing depressive symptoms in the general population across the adult lifespan. Scores range from 0 to 63 with the following cutoffs: minimal depression 0–13, mild depression 14–19, moderate depression 20–28, severe depression > 29. BDI-II scores in the sample ranged from 0 to 39. Only 11 of the enrolled participants had scores in the moderate or severe range supporting a range of depression levels and subthreshold depression levels for the bulk of the sample. These neuropsychological assessments and structural imaging (see below) were completed in separate visits within a 1-month period. Our cognitive domain of interest in this study was executive functioning. However, only 158 subjects were also administered the other test of executive functioning, the Color-Word Stroop. Consequently, we focused on the Trail Making Test as our measure of executive functioning. Specifically, as has been used in prior studies (Bickford et al., 2018; Dulas & Duarte, 2016; Enright et al., 2015), we subtracted transformed Trails A reaction time from transformed Trails B reaction time, z-scored the difference scores and used them as dependent variables in regression analyses described below.

### Structural MRI acquisition and analysis

Scanning was performed on a 3Т Siemens Skyra scanner at the UT Austin Brain Imaging Center. A high-resolution T1-weighted magnetization prepared rapid gradient echo (MPRAGE) image was collected (TR = 2530 ms, TE = 30 ms, 256 × 256 matrix, 42 axial slices, 1 mm slice thickness, no gap).

Preprocessing and statistical analyses of structural imaging data were performed using Statistical Parametric Mapping software for MATLAB (SPM 12, Wellcome Department of Cognitive Neurology, London, UK). For voxel-based morphometry (VBM) analyses, the T1-weighted MRI scans were bias-corrected, segmented into gray matter, white matter and cerebral spinal fluid components. We used the Diffeomorphic Anatomic Registration Through Exponentiated Lie algebra algorithm (DARTEL) toolbox in order to generate a study-specific template for the segmented 3D data. Segmented, modulated, grey matter images were normalized into standard Montreal Neurological Institute (MNI) space and smoothed with an 8-mm full-width-half-maximum (FWHM) Gaussian smoothing kernel. Intracranial volume (ICV) was calculated with the SPM12 automated Module Tissue Volumes (Ashburner, 2010).

### Region of interest (ROI) definition

**Fig. 1:**
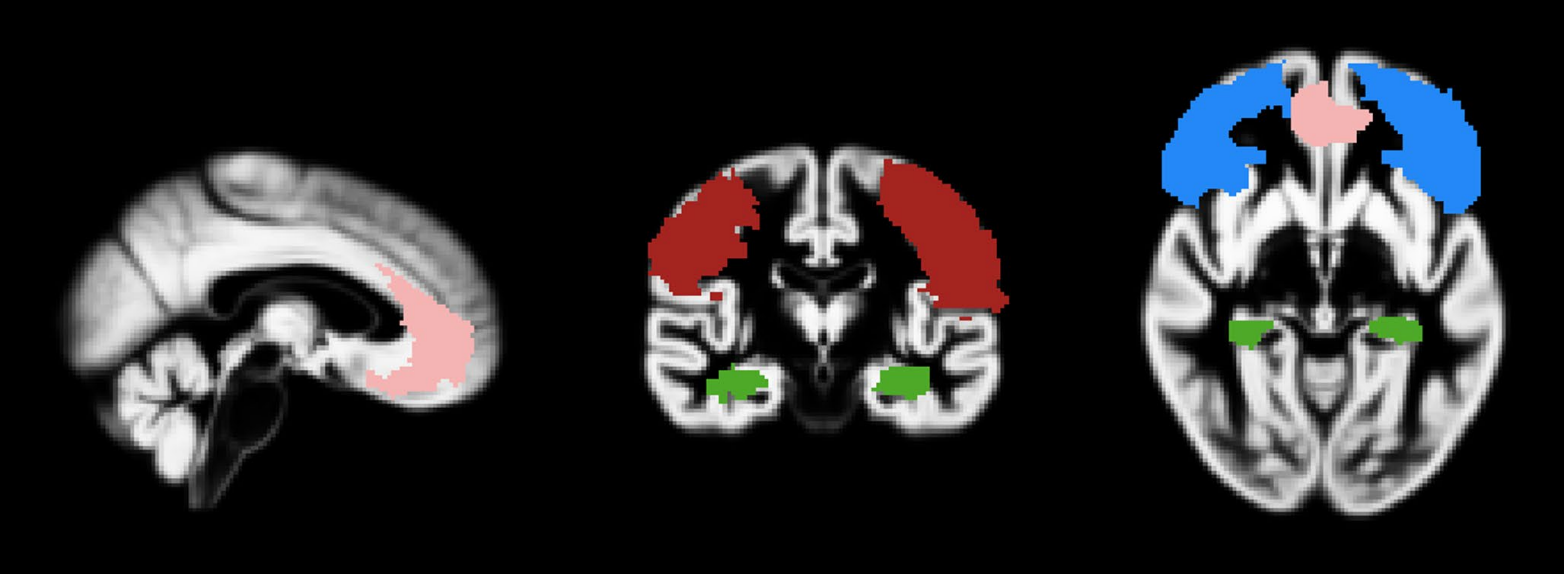
Realigned ROI masks shown on the group template brain: ACC shown in pink, OFC shown in blue, LPFC shown in red, Hippocampus shown in green

Three bilateral regions of interest (ROIs) were generated from the Anatomical Automatic Labeling (AAL) system (Tzourio-Mazoyer et al., 2002) implemented in the WFU Pick Atlas software toolbox (Maldjian et al., 2003): Three ROIs were selected, all located in PFC, (1) orbitofrontal cortex (OFC), consisting of superior orbital frontal, inferior orbital frontal, and medial orbital frontal gyri; (2) lateral prefrontal cortex (LPFC) including middle and inferior frontal gyri; (3) and the anterior cingulate cortex (ACC) as shown below in Fig. 1. Volumes of these ROIs were extracted using get_total.m (Ridgway, 2014) imported into RStudio for further analyses.

We also investigated the hippocampus as an ROI as in previous studies, evidence has shown that smaller hippocampus volume is associated with poorer episodic memory, executive function performance, and depression in older ages (Hardcastle et al., 2020; James et al., 2021).

### Hierarchal regression analysis

Hierarchal regression analyses were performed using R studio. For all regression models, in step 1, we included sex, years of education, chronological age, and race as predictors (adding intracranial volume for models with brain volume outcomes). We added depression level, measured with the BDI, as a predictor in step 2. The interaction between age and BDI was added in step 3. Executive functioning scores, and each of the ROI volumes served as the outcome variables in separate models. We’ve conducted multiple testing corrections for all regression analysis with FDR (Benjamin-Hochberg tests) adjusted p values reported.

### Mediation analysis

**Fig. 2:**
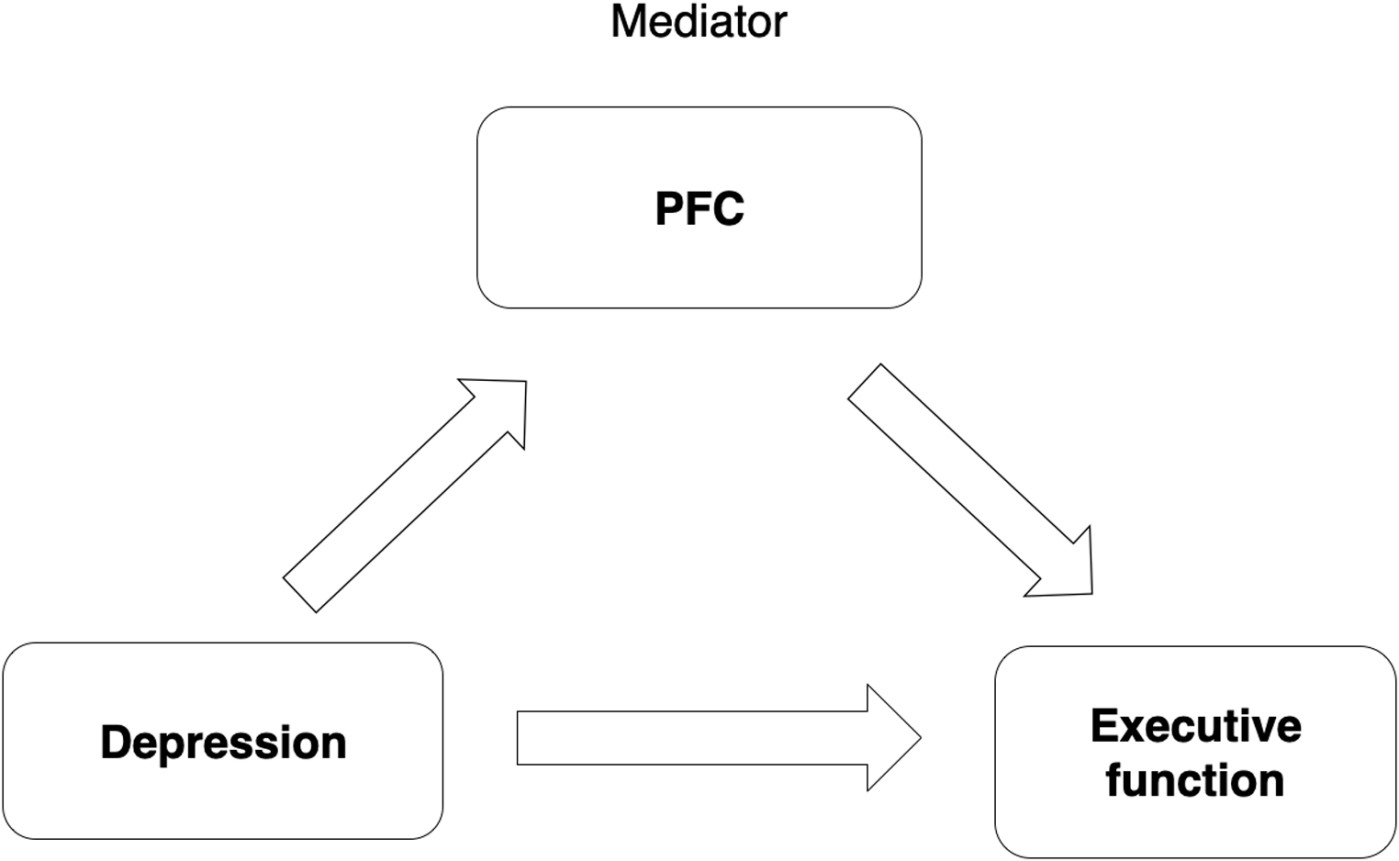
Mediation model

In order to explore potential mediators of the relationship between depression and cognitive abilities and/or brain volumes, we included bilateral PFC brain volumes for each ROI as a mediator of relationships, should they be observed, in RStudio (Fig. 2). We tested the total, direct and indirect influence of PFC volumes in the whole sample. We used bootstrapping with 1000 simulations to generate a sampling distribution and test the statistical significance of the effects. Moderated mediation with race as moderator was also conducted in R studio with model 59 from PROCESS (Hayes, 2022) in order to explore whether mediations differed by racial group.

## Results

Correlations between the study variables are shown in Table 2. As can be seen in the table, across subjects, depression severity scores were negatively correlated with LPFC and ACC volumes and executive functioning scores were positively correlated with LPFC, ACC, and OFC volumes. Note that a larger executive functioning score indicates slower reaction times and worse executive functioning.

**Table 2:** Descriptive statistics and correlation coefficient of study variables (*N* = 176)

Variables	1	2	3	4	5	6	7	8
1.Race	-							
2.Sex	0.07	-						
3.Years of Education	-0.190*	-0.102	-					
4.Depression	-0.0025	-0.02	-0.099	-				
5.Age	-0.032	-0.145	0.0786	0.073	-			
6. Executive Function	0.215**	0.038	-0.199**	0.244**	0.0299	-		
7.LPFC	-0.246**	-0.268**	0.016	-0.136	0.005	-0.27**	-	
8.OFC	-0.347**	-0.398**	0.152	-0.11	0.074	-0.299**	0.822**	-
9.ACC	-0.334**	-0.366**	0.149	-0.176*	0.071	-0.26**	0.768**	0.875**

*Note* Race (0 = Non-Hispanic White, 1 = Black & Hispanic American); Gender (0 = Male, 1 = Female). *p* < 0.05 *, *p* < 0.01**

### Depression and executive functioning

The results of the hierarchical regression model with executive functioning score as the outcome are shown in Table 3. As can be seen in the table, higher depression scores, minoritized racial and ethnic status, and fewer years of education were significantly related to lower executive functional ability (larger RT) across groups. For example, an increase of 1 point on the CESD-R is associated with an increase of 0.038 (beta coefficient (***B***)) z score in executive dysfunction. The 3-way interaction between depression, race and age was significant and survived multiple testing correction.

**Table 3:** Hierarchical regression with age, race, and depression predicting executive functioning

	Model 1			Model 2			Model 3		
	*B*	*SE B*	*β*	*B*	*SE B*	*β*	*B*	*SE B*	*β*
Education	-0.075	0.034	-0.167	-0.063	0.033	-0.141	-0.066	0.0312	-0.147
Sex	0.034	0.169	0.015	0.043	0.165	0.019	0.021	0.155	0.009
Age	0.009	0.013	0.051	0.006	0.013	0.0331	-0.009	0.02	-0.054
Race	0.434	0.177	0.184	0.445	0.173	0.189	3.624	1.786	1.536
Depression	—	—	—	0.038*	0.012	0.228	-0.033	0.106	-0.196
Age x Depression	—	—	—	—	—	—	0.001	0.002	0.349
Race x Depression	—	—	—	—	—	—	-0.64*	0.18	-3.106
Age x Depression x race	—	—	—	—	—	—	0.014*	0.004	-1.501
*R*2		0.075			0.126			0.262	
*F* for Δ*R*2		3.465			9.951			7.615	

*Note* ***β*** reflect standardized coefficients, which are unitless, and are interpreted as the number of standard deviation change in the outcome per every standard deviation increase in the predictor. * reflects significant tests for FDR (Benjamin-Hochberg tests) adjusted *p* values

**Fig. 3:**
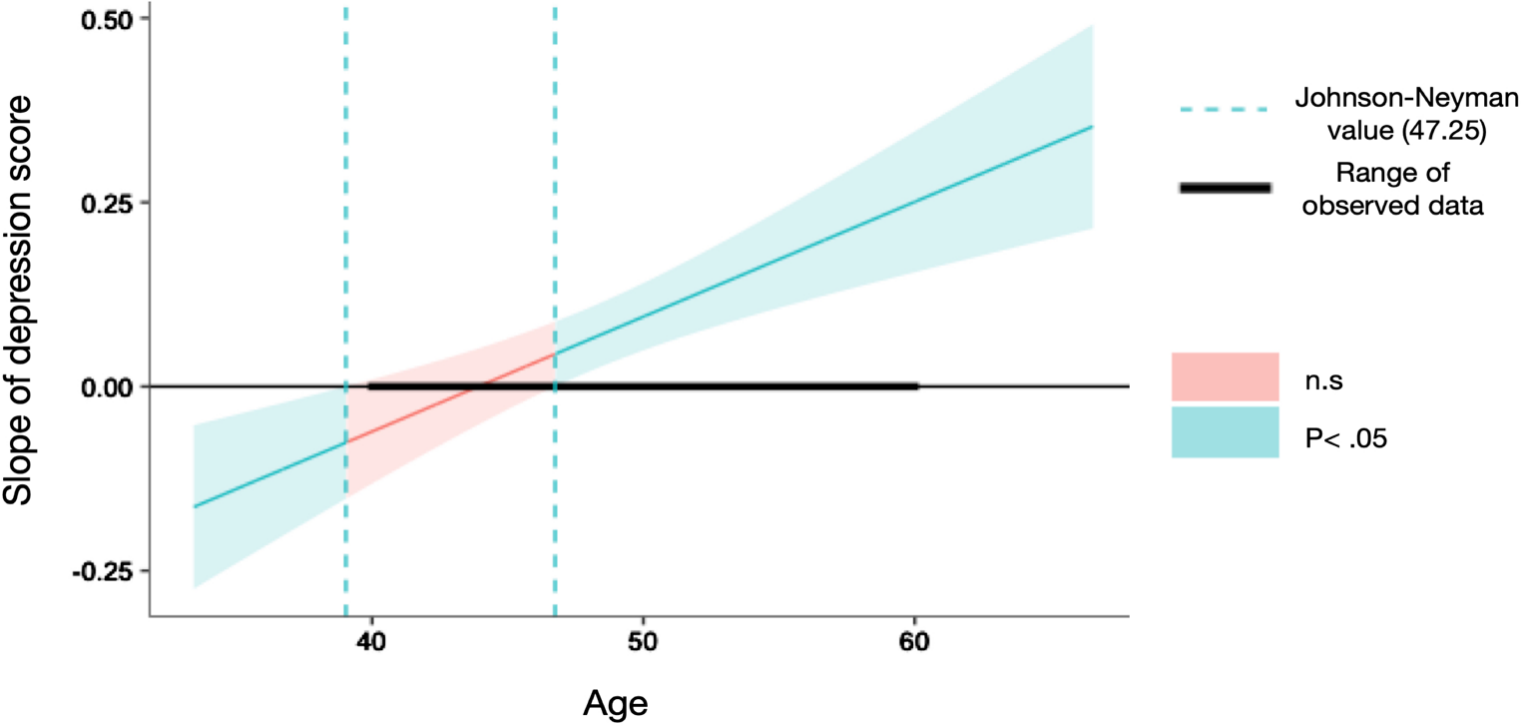
Age x depression interaction on executive functioning for the Black & Hispanic American group Johnson-Neyman value (46.75) *Note*: The dashed line is the Johnson-Neyman value (age = 46.75), indicating the slope over 46.75 is significantly from zero. The line represents slope coefficients of depression score on executive functioning, across age. The shaded area refers to the 95% confidence interval

To explore the source of the interactions including Race, we conducted regression analyses separately for non-Hispanic White and Black & Hispanic American groups. The interaction between Age and Depression was significant after multiple test correction (see Method) in the Black and Hispanic American group (*B* = 0.016, *F* = 19.616, *R*^*2*^ = 0.226, *p* = 0.0002) but not the Non-Hispanic White group (*B* = 0.001, *F* = 0.306, *R*^*2*^ = 0.0023, *p* = 0.9134). As can be seen in Fig. 3, the Johnson-Neyman significance region for depression score as a predictor of executive function was beyond age 46.75 within the observed data age interval of 40 to 60. In other words, for the Black & Hispanic American group only, a higher depression score was significantly predictive of lower executive functioning as age increased.

We conducted the same analysis using Color-Word Stroop accuracy in word/color proactive interference condition, but found no effects apart from race, indicating lower accuracy in the Black and Hispanic Group (***B*** =-3.114, F = 0.963, *R*^*2*^= 0.02995, *p* = 0.467).

### Depression and brain volume

We conducted the same hierarchical regression model as above with depression as a predictor of PFC volumes (see Supplemental Material for regression tables). Higher depression scores were associated with smaller ACC volumes across age and racial groups (*B*= -0.031, F = 19.62, *R*^*2*^ = 0.411, *p* = 0.012) but not LPFC (*B*=-0.0931, F = 9.391, *R*^*2*^ = 0.25, *p* = 0.138) or OFC (*B*= -0.038, F = 24.91, *R*^*2*^ = 0.469, *p* = 0.15) volumes after multiple test corrections. Race was a significant predictor of OFC and ACC volumes, with smaller volumes in the Black and Hispanic adult group than the non-Hispanic White adult group (*B’s* < -0.417, *p’s* < 0.03). No other predictors or interactions were significant (all *B’s* < 0.024, all *p’s* > 0.6). No significant effects involving any predictors, except for ICV, were observed for the hippocampus (see Supplemental Material).

## Mediation analysis

**Fig. 4:**
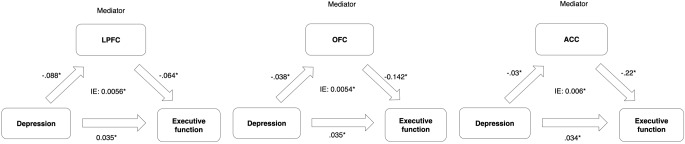
Mediation model results of depression predicting executive function with PFC volumes as mediators *Note.* Values reflected path coefficients in the model, with * showing significance

To explore the underlying mechanisms of the relationship between depression and executive functioning, we conducted mediation analyses to examine whether PFC brain volumes mediate this relationship, across race and age, in order to increase statistical power. As can be seen in Fig. 4, there were significant indirect effects between depression and executive functioning through each of the PFC volumes. Direct effects for each model remained significant, suggesting that PFC volumes were partial mediators of the relationship between depression and executive functioning. Moderated mediation analysis with racial group as moderator shows indirect effects were not conditional upon race for any PFC volumes, indicating that PFC volumes partially mediated depression-executive functioning relationships similarly across groups (Conditional indirect effects: LPFC [BootLLCI:-0.007, BootULCI:0.03] OFC [BootLLCI:-0.003, BootULCI:0.043], ACC [BootLLCI:-0.008, BootULCI:0.02]).

Mediation analysis conducted with the hippocampus volume showed no significant direct or indirect effects (Direct effect: [BootLLCI:-0.555, BootULCI:0.21]; Conditional indirect effects: [BootLLCI:-0.011, BootULCI:0.012]).

## Discussion

Executive dysfunction is one of the primary cognitive impairments in MDD and depressive symptoms, even in the absence of diagnosed MDD, are associated with worse performance on numerous tests of executive functioning (i.e., Trails, Stroop, and Flanker) in adults across the lifespan (Dotson et al., 2020). Numerous studies have shown under-recruitment of lateral PFC regions during the performance of executive functioning tasks in MDD compared to non-depressed adults as well as depression-related volume reductions in lateral and orbitofrontal (e.g., Hurley & Tizabi, 2013; Pizzagalli & Roberts, 2022). Our results showing depression-associated executive functioning impairments, which were partially mediated by reduced lateral, orbitofrontal, and ACC volumes are consistent with this literature. Our results add to a growing literature showing that even minimal depressive symptoms, in the absence of MDD, may predict executive dysfunction and extend these findings to middle-aged adults (Dotson et al., 2009; Li et al., 2017).

Importantly, our results extend this prior work showing ethnoracial moderations of these relationships. Specifically, greater depressive symptom severity was associated with worse executive functioning in Black and Hispanic adults, particularly with increasing age (i.e., > 46 years). The same age moderation was not observed in non-Hispanic White adults. Emerging work has shown that depression-related cognitive impairments may be magnified in older age. For example, recent meta-analyses, including from our group, have shown that depression-related impairments in tests of episodic memory (James et al., 2021) and executive functioning (Dotson et al., 2020) are magnified in older age, particularly in later decades. Given that PFC volume reductions are well-established in both cognitively unimpaired aging (Raz, 2000; Raz & Rodrigue, 2006) and depression (e.g., Hurley & Tizabi, 2013; Pizzagalli & Roberts, 2022), it follows that age may magnify depression-related executive dysfunction.

Prior studies are limited, however, in a relative lack of middle-aged adults with most studies focusing on young or older subjects, and a lack of racial and ethnic diversity of research subjects. Our results fill in this gap by showing that executive dysfunction is subject to synergistic effects of depressive symptom severity and age even within middle age. Importantly, this synergistic effect was only significant in the ethnoracial minority group. Although few studies that have assessed depression-related cognitive impairment in Blacks and Hispanics, emerging research has shown that depressive symptoms may have greater consequences for these individuals than for non-Hispanic White adult. For example, moderate executive functioning impairments have been observed for a number of executive functioning tasks in these minority groups compared to non-Hispanic White adults across the adult lifespan, with little difference between Blacks and Hispanics adults (Rea-Sandin et al., 2021). Some evidence suggests that depressive symptoms are more strongly related to executive dysfunction in Black compared to non-Hispanic White older adults (Zahodne et al., 2014). Here, we show that even in middle-age, mild depressive symptoms may have more severe consequences in ethnoracial minorities, effectively moving forward the point at which age-related executive functioning impairments appear. While the statistical effect size was large, the increase in level of executive dysfunction (z-score for RT slowing on Trails B-A) for each standard deviation increase in level of depression severity was relatively small (***B*** = 0.038). However, given that this relationship was observed in middle-aged adults without any clinically significant cognitive impairments, this seemingly subtle change in performance it is not particularly surprising. Nonetheless, as depression has been associated with a greater risk of cognitive decline in Black and Hispanic individuals than age-matched, older, non-Hispanic White adults (Abrams & Mehta, 2019; Babulal et al., 2022; Barnes & Bates, 2017; Vyas et al., 2020), these results suggest that depressive symptoms in middle age may be an early indicator of subsequent cognitive decline specifically in Black and Hispanic individuals.

## Limitations, clinical implications, and future directions

This study has a few limitations. The sample is relatively small, especially the historically minoritized sample, although this did not preclude our ability to detect significant associations between our factors of interest. Nonetheless, it will be important for future studies to replicate these effects in larger samples and to separate the subsamples of the minoritized group. We did not observe any associations between depression symptom severity and hippocampal volumes, in contrast to prior evidence (James et al., 2021). The most likely explanation for these null effects is the restricted range of performance on the episodic memory measure, where participants, across groups, performed very well. It is also plausible that hippocampal volumes, which show the greatest evidence of age-related volume loss in later decades (Salami et al., 2012), were insensitive either age or depression level in the middle-aged sample in this study.

This study used an archival dataset in which some of the variables of interest were missing for some participants. In order to avoid introducing bias related to overfitting from multiple imputation methods, which could be greater in small samples, we excluded subjects missing critical variables including age, race/ethnicity, BDI or cognitive scores. However, a limitation of removing subjects from analysis is the potential introduction of a sampling bias. Furthermore, our ability to explore other potential mediating factors of the relationship between depression level and executive dysfunction was limited by the use of the archival data set. For example, some evidence shows that life stress is related to depression, and ethnoracial minorities are more likely to experience these conditions than non-Hispanic White adults (Kim & Cho, 2020; Zuelsdorff et al., 2020). It is possible, for example, that these or other factors contributed to the smaller PFC volumes in the ethnoracial minority group compared to the non-Hispanic White adults in this study, which in turn contributed to depression-related executive dysfunction. Some of these psychosocial and health factors may be bidirectionally related to depression. For example in prospective studies, As described in the vascular depression hypothesis (González & Tarraf, 2013; Joynt et al., 2003; Taylor et al., 2013), evidence of cardiovascular disease (CVD) (e.g. coronary heart disease) has been shown to predict later emergence of major depression; and depression has been identified as an independent predictor of risk of CVD, with evidence of dose-response relationships (Serrano et al., 2011). Longitudinal assessments with repeated measurements of CVD and depressive symptomology would be needed to better understand the directionality of this relationship in the minoritized population, which could have important clinical implications. For example, individuals with high depression severity but no evidence of CVD, which likely includes some of the subjects in the current study, could benefit from depression treatment that could, in turn, delay or prevent development of CVD through a reduction of stress-related inflammation. It will be important to explore the extent to which these various individual difference factors, especially modifiable ones like CVD, explain racial and ethnic group differences in depression-related impairments.

This work also has implications for disorders in which mood disruption and/or cognitive impairments are observed, such as Alzheimer’s disease. Our inclusion of an ethnoracially diverse sample enhances the generalizability of our results to groups who are at a greater risk of dementia (Mehta & Yeo, 2017).

That is, depressive symptoms in middle aged and older individuals may be a risk factor for cognitive decline and Alzheimer’s disease (Gale et al., 2012; Wilson et al., 2014), rates of which are higher in Black and Hispanic than Non-Hispanic White adults (Matthews et al., 2019). Thus, clinicians may want to more closely monitor even mild depressive symptoms in patients, especially those from these historically minoritized groups. Our examination of depressive symptoms as a spectrum of severity also maximizes the applicability of results since subthreshold depressive symptoms are more common than clinical depression (Biella et al., 2019).

## Conclusion

This pilot study aims to examine the potential moderating influence of age and mediating influence of PFC volume on relationships between depressive symptoms and executive functioning in historically minoritized, namely Black and Hispanic adults, and non-Hispanic white middle-aged adults. We predicted that individuals with greater levels of depressive symptoms would show lower executive function scores and smaller PFC brain volumes and tested the novel hypotheses that age would exacerbate these associations particularly in the minoritized group. Our results were largely consistent with these predictions. We found that depression predicted worse executive functioning across groups, with age exacerbating this relationship exclusively in the Black and Hispanic adults group. PFC volumes, which were reduced in the Black and Hispanic adults compared to the non-Hispanic white adults partially mediated the relationship between depression level and executive functioning, across age and ethnoracial group.

## Electronic supplementary material

Below is the link to the electronic supplementary material.

Supplementary Material 1

## Data Availability

The dataset is available in Open Science Framework via https://osf.io/qancx/?view_only=d91f8c8f539c4cb79629208aa62027b4. DOI 10.17605/OSF.IO/QANCX.
