# Supplementary material for: Age exacerbates the negative effect of depression on executive functioning in racial and ethnic minorities
Source: Brain Imaging Behav. 2024 Jun 8;18(5):1064–74. doi: 10.1007/s11682-024-00898-3 (PMC11582303; doi:10.1007/s11682-024-00898-3)
Supplement: Supplementary file 1 — Supplementary Material 1 [file 11682_2024_898_MOESM1_ESM.docx]

**Supplement**

**Table 4.** Hierarchical regression with depression predicting LPFC brain volumes

| LPFC | **Model 1** | | | **Model 2** | | |
| --- | --- | --- | --- | --- | --- | --- |
|  | ***B*** | ***SE B*** | ***β*** | ***B*** | ***SE B*** | ***β*** |
| Education | -0.121 | 0.117 | -0.072 | -0.15 | 0.116 | -0.088 |
| Sex | -0.321 | 0.71 | -0.038 | -0.338 | 0.702 | -0.04 |
| Age | -0.022 | 0.045 | -0.033 | -0.014 | 0.044 | -0.022 |
| Race | -1.18 | 0.643 | -0.133 | -1.203 | 0.636 | -0.135 |
| ICV | 11.971* | 2.549 | 0.408 | 12.007* | 2.52 | 0.409 |
| Depression | — | — | — | -0.0931 | 0.0491 | -0.149 |
| *R*2 |  | 0.2281 |  |  | 0.25 |  |
| *F* for Δ*R*2 |  | 10.05 |  |  | 4.93 |  |

**Note.** * reflects significant tests for FDR (Benjamin-Hochberg tests) adjusted p values.

**Table 5.** Hierarchical regression with depression predicting OFC brain volumes

| OFC | **Model 1** | | | **Model 2** | | |
| --- | --- | --- | --- | --- | --- | --- |
|  | ***B*** | ***SE B*** | ***β*** | ***B*** | ***SE B*** | ***β*** |
| Education | -0.026 | 0.051 | 0.03 | 0.015 | 0.05 | 0.017 |
| Sex | -0.262 | 0.307 | -0.06 | -0.269 | 0.305 | -0.061 |
| Age | 0.004 | 0.019 | 0.0109 | 0.007 | 0.019 | 0.02 |
| Race | -0.749 | 0.278 | -0.163 | -0.758* | 0.276 | -0.166 |
| ICV | 8.545* | 1.105 | 0.565 | 8.56* | 1.094 | 0.566 |
| Depression | — | — | — | -0.038 | 0.018 | -0.118 |
| *R*2 |  | 0.456 |  |  | 0.469 |  |
| *F* for Δ*R*2 |  | 28.45 |  |  | 4.3779 |  |

**Note.** * reflects significant tests for FDR (Benjamin-Hochberg tests) adjusted p values.

**Table 6.** Hierarchical regression with depression predicting ACC brain volumes

| ACC | **Model 1** | | | **Model 2** | | |
| --- | --- | --- | --- | --- | --- | --- |
|  | ***B*** | ***SE B*** | ***β*** | ***B*** | ***SE B*** | ***β*** |
| Education | 0.016 | 0.028 | 0.035 | 0.007 | 0.028 | 0.015 |
| Sex | -0.157 | 0.171 | -0.069 | -0.163 | 0.167 | -0.072 |
| Age | 0.002 | 0.011 | 0.012 | 0.005 | 0.011 | 0.026 |
| Race | -0.41 | 0.155 | -0.172 | -0.417* | 0.151 | -0.175 |
| ICV | 3.849* | 0.613 | 0.49 | 3.861* | 0.598 | 0.492 |
| Depression | — | — | — | -0.031* | 0.0099 | -0.184 |
| *R*2 |  | 0.377 |  |  | 0.411 |  |
| *F* for Δ*R*2 |  | 20.61 |  |  | 9.501 |  |

**Note.** * reflects significant tests for FDR (Benjamin-Hochberg tests) adjusted p values.

**Table 7**. Hierarchical regression with depression predicting Hippocampus volumes

|  | **Model 1** | | | **Model 2** | | |
| --- | --- | --- | --- | --- | --- | --- |
| Hippocampus | ***B*** | ***SE B*** | ***β*** | ***B*** | ***SE B*** | ***β*** |
| Education | -0.013 | 0.014 | -0.057 | -0.013 | 0.014 | -0.059 |
| Sex | -0.194 | 0.086 | -0.165 | -0.194 | 0.086 | -0.013 |
| Age | 0.004 | 0.005 | 0.045 | 0.004 | 0.005 | 0.046 |
| Race | -0.015 | 0.078 | -0.013 | -0.0154 | 0.078 | -0.013 |
| ICV | 2.135* | 0.309 | 0.527* | 2.135* | 0.309 | 0.527* |
| Depression | — | — | — | -0.0016 | 0.005 | -0.019 |
| *R*2 |  | 0.408 |  |  | 0.409 |  |
| *F* for ΔR2 |  | 23.44 |  |  | 0.0992 |  |

**Note:** * reflects significant tests for FDR (Benjamin-Hochberg tests) adjusted p values.
